# Assessing the evolution of wheat grain traits during the last 166 years using archived samples

**DOI:** 10.1038/s41598-020-78504-x

**Published:** 2020-12-11

**Authors:** Sinda Ben Mariem, Angie L. Gámez, Luis Larraya, Teresa Fuertes-Mendizabal, Nuria Cañameras, José L. Araus, Steve P. McGrath, Malcolm J. Hawkesford, Carmen Gonzalez Murua, Myriam Gaudeul, Leopoldo Medina, Alan Paton, Luigi Cattivelli, Andreas Fangmeier, James Bunce, Sabine Tausz-Posch, Andy J. Macdonald, Iker Aranjuelo

**Affiliations:** 1 Spanish National Research Council (CSIC)-Government of Navarre, AgroBiotechnology Institute (IdAB), Av. Pamplona 123, 31006 Mutilva, Spain; 2 Institute for Multidisciplinary Applied Biology, Dpto. Agronomía, Biotecnología y Alimentación, Universidad Pública de Navarra, Campus Arrosadia, 31006 Pamplona, Spain; 3 Department of Plant Biology and Ecology, University of the Basque Country (UPV/EHU), Bilbao, Spain; 4 Universitat Politècnica de Catalunya, EsteveTerrades 8, Building 4, Castelldefels, Spain; 5 Integrative Crop Ecophysiology Group, Plant Physiology Section, Faculty of Biology, University of Barcelona, Barcelona, and AGROTECNIO Center, Lleida, Spain; 6 Sustainable Agriculture Sciences, Rothamsted Research, Harpenden, AL5 2JQ Hertfordshire UK; 7 Plant Sciences, Rothamsted Research, Harpenden, AL5 2JQ Hertfordshire UK; 8 Institut de Systématique, Évolution, Biodiversité (ISYEB), Muséum National D’Histoire Naturelle, CNRS, EPHE, UA, Sorbonne Université, 57 rue Cuvier, CP 39, 75005 Paris, France; 9 Spanish National Research Council (CSIC), Real Jardín Botánico, C/ Claudio Moyano 1, Madrid, Spain; 10 Royal Botanic Gardens Kew, Kew Richmond, TW9 3AB UK; 11 Agricultural Research Council (CREA), Centre for Genomics and Bioinformatics, Via San Protaso 302, Fiorenzuolad’Arda, Italy; 12 Institute of Landscape and Plant Ecology, University of Hohenheim, August-von-Hartmann-Str. 3, 70599 Stuttgart, Germany; 13 Adaptive Cropping Systems Lab (Retired), Beltsville Agricultural Research Center, Agricultural Research Service, US Department of Agriculture, Beltsville, MD 20705 USA; 14 Department of Agriculture, Science and the Environment, School of Health, Medical and Applied Sciences, CQ University Australia, Rockhampton, QLD Australia

**Keywords:** Natural variation in plants, Plant development, Plant stress responses

## Abstract

The current study focuses on yield and nutritional quality changes of wheat grain over the last 166 years. It is based on wheat grain quality analyses carried out on samples collected between 1850 and 2016. Samples were obtained from the Broadbalk Continuous Wheat Experiment (UK) and from herbaria from 16 different countries around the world. Our study showed that, together with an increase in carbohydrate content, an impoverishment of mineral composition and protein content occurred. The imbalance in carbohydrate/protein content was specially marked after the 1960’s, coinciding with strong increases in ambient [CO_2_] and temperature and the introduction of progressively shorter straw varieties. The implications of altered crop physiology are discussed.

## Introduction

Environmental changes, including climate change, land degradation and biodiversity loss, have been particularly apparent in recent decades and are predicted to become even more extreme in the twenty-first century^1^. The environmental conditions in which plants have been growing during the last century have changed considerably since the Industrial Revolution^2^. The atmospheric carbon dioxide concentration [CO_2_] has increased from about 280 ppm in pre-industrial times to 406 ppm reached in 2017^3^. A direct consequence of the elevated atmospheric [CO_2_] (and other greenhouse gas concentrations) is the increase in global temperature and evaporative water demand, paralleled by a reduction in water availability in many regions. It is reported that the average global and ocean surface temperature has increased by approximately 0.85 °C [0.65 to 1.06 °C] over the period 1880 to 2012^2^. These changes have affected agriculture globally and will create significant challenges for food security and nutrition in the future. Indeed, as observed by Oury et al.^4^, the beneficial effects expected from the increase in [CO_2_] in European crop production during recent decades have been constrained by the effects of temperature increases and extended drought periods. This is a major issue, because cereal grains are a key source of carbohydrates, proteins, amino acids, lipids, vitamins and minerals, all of which determine the nutritional value and quality of wheat products^5^. More specifically, those crops provide 44% of the daily dietary intake of Fe, 27% of Mg, 25% of Zn and 31% of Cu^6^. Further, wheat is the second most important food crop after rice, and provides 20% of the daily protein and food calories worldwide^7^.

Environmental conditions have a significant impact on crop production. Since the current atmospheric [CO_2_] is generally limiting for plants with photosynthetic C_3_ metabolism, the available information suggests that increases in [CO_2_] should contribute to increased crop growth and yield^8,9^. Alongside changes in crop yield, other studies carried out during the last few decades^10,11^ have revealed that exposure to increasing [CO_2_] causes alterations to the mineral content of plant tissues, total protein concentration and lipid composition. Within this context, it has been suggested that because grains are predominantly composed of carbohydrates (mostly in the form of starch), the expected increases in starch concentrations due to the high [CO_2_] could dilute other nutrients, including proteins, lipids, vitamins, and minerals^12,13^.

Most plant studies are based on experiments in controlled environment (such as growth chambers) or field conditions, in which [CO_2_] concentrations were fixed at 550–700 ppm. According to the different scenarios proposed by the IPCC^2^, these may be the ambient [CO_2_] values that will be reached by 2050 and 2100, respectively. Nevertheless, new approaches to field studies have been developed. These include free air systems, such as the free air [CO_2_] enrichment (FACE), controlled [CO_2_] greenhouses (CGH), or open-top chambers (OTC)^14,15^. It should be noted that, according to Högy et al.^16^, the high [CO_2_] stimulation effect might be larger in growth chambers and glasshouses compared to field exposure. While FACE overcomes many of the disadvantages associated with chamber and glasshouse experiments, some potential limitations have been attributed to these facilities when simulating natural growth conditions. A number of challenges exist when conducting FACE experiments, including the difficulty of managing large numbers of sites, replication within sites, physiological impact of a relatively abrupt increase in [CO_2_], effect of CO_2_ influx on breaking up an inversion layer, and consequent impact on temperature fluctuations within the open-top chamber^17,18^. Consequently, while FACE experiments approach natural conditions more closely than open-top chambers or other means of exposing plants to elevated [CO_2_], they may still represent an approximation of natural growing conditions, albeit a method that is close to natural growth conditions. This may be a major issue to consider because the more realistic the experimental conditions, the more likely it is that the resultant predictions will reflect the reality of the future. However, few studies have addressed the effect of lower-than-present [CO_2_] on grain quality and to the best of our knowledge, all of them have been performed in growth chambers simulating past conditions. As an alternative, the analyses of old samples from herbaria and other repositories should allow direct assessment of the effect of past growing conditions on contemporary samples from crops that were grown at that time. Within this context, in the last two decades, several studies have highlighted the use of archived material to analyse the changes in plant mineral composition over recent decades^6,19^. However, herbarium material has often insufficient information about the location and environmental conditions where the plants were grown or the cultivars used.

The data presented in this paper aims to evaluate the impact of long-term changes in atmospheric [CO_2_], temperature and rainfall conditions on wheat grain quality traits (carbohydrates, protein and mineral concentration) in archived samples during the last 166 years and the association between such quality traits and increased yield.

## Material and methods

### Grain material collection

In this study, bread and durum wheat grains were collected from archives of 16 countries around the world (Table 1), from 1850 to 2016. All grain samples selected were intact, without any visible signs of degradation. One part of the archived grain samples originated from the Broadbalk Wheat experiment (Rothamsted, UK). They were taken from plots given annual applications of Farmyard Manure (35 tha^−1^ fresh material) since 1843. Another part of the samples (called the ‘global samples’) originated from 16 countries, and were removed from herbarium specimens stored at the Universitat Politecnica de Catalunya (Castel ldefels, Spain), Muséum National d’Histoire naturelle (Paris, France), Real Jardín Botánico (Madrid, Spain) and Royal Botanic Gardens (Kew Richmond, United Kingdom).

**Table 1:** Geographic origin and sampling year of archived wheat grain samples.

Continent	Country	Location	Sampling year	Number of replicates per year
Eurasia	Spain	Albacete	1900	3
		Lleida	1900	3
		Logroño	1850	3
		Madrid	1920	3
		Lugo	1953	3
		Segovia	1985	3
		Valladolid	1985	3
		Serrania de Ronda	1905	3
		Aranjuez	2015	3
		Cordoba	2015	3
		Zamora	2015	3
	United Kingdom	Rothamsted	[1850–2016]	3
		Regent Road, Leicester	1944	3
		City of London, Middlesex	1945	3
	New Caledonia	No data	1937	3
		Pouembout	1965	3
	Italy	Tuscany	1883	3
		Fiorenzuola d’Arda	2016	14
	Serbia/UK	Suva plains but cultivated at Royal Botanic Gardens Kew	1923	3
	Germany	Hohenheim	2016	3
	Russia	No data	1900	3
	No data (Asia)	No data	1910	3
Africa	Algeria	Oran	1856	3
	Nigeria	No data	1921	3
South America	Argentina	No data	1900	3
North America	USA	No data	1900	3
		Beltsville	2016	3
Asia (Near East)	Iraq	Zakh-Mosul	1947	3
		Mariye 4 km NW of Rawa	1947	3
	Iran	locality illegible	1885	3
	Yemen	Wadi Hadhramant	1946	6
Asia	China	No data	2016	6
Oceania	Australia	No data	2016	8

The Broadbalk experiment is the oldest continuous agronomic field experiment in the world, which started in 1843. This 176 year-old experiment provides a large number of archived crop and soil samples from a wide range of agricultural, environmental and ecological conditions. Grain yield data (1850–2016) were available for the Broadbalk experiment, but not from the other sites used in this study. Wheat varieties analyzed in the current study are shown in Table S1. In addition, thousand kernel weights (TKW) were available for Broadbalk from 1974 until 2016. In the early years (1844–1901), the crop from each plot was cut by hand with scythes, bound into sheaves and carted into the barns to await threshing. Broadbalk is now harvested by a small plot combine harvester with a 2 m cut width. Yields of grain and straw are recorded, and samples stored for chemical analyses.

### Environmental [CO_2_], temperature and precipitation data between 1850 and 2016

Global atmospheric [CO_2_] values (Table 2) for the period from 1850 to 2016 were obtained from the European Environment Agency web page^3^. Data corresponding to the evolution of ambient temperature were extracted from the Intergovernmental Panel on Climate Change^20^. Average temperature and precipitation recorded at Rothamsted (Table 2) were provided by the Department of Computational and Analytical Sciences.

**Table 2:** Average environmental data recorded from 1850 to 2016.

Area	[CO_2_] (ppm)		Temperature (°C)		Precipitation (mm)	
	Years	Values	Years	values	Years	values
Global	1850	286	1850	13.7	No data	
	1856	286	1860	13.6		
	1883	293	1870	13.8		
	1885	293	1880	13.9		
	1900	297	1890	13.7		
	1905	299	1900	13.8		
	1910	299	1910	13.5		
	1921	303	1920	13.7		
	1923	305	1930	13.9		
	1937	308	1940	14.0		
	1947	310	1950	13.8		
	1953	312	1960	13.9		
	1955	314	1970	13.9		
	1965	318	1980	14.0		
	1975	331	1990	14.2		
	1985	345	2000	14.4		
	1990	352	2010	14.7		
	2000	378	2016	14.9		
	2010	390				
	2016	400				
Rothamsted	1850	286	1878	9.28	1878	814.2
	1900	297	1900	9.37	1900	715.8
	1955	314	1955	8.99	1955	592.1
	1975	331	1975	9.60	1975	612.3
	1990	352	1990	10.25	1990	597.4
	2000	378	2000	10.20	2000	973.5
	2010	390	2010	9.01	2010	644.2
	2016	405	2016	10.35	2016	679.3

### Grain quality parameters

#### Carbon isotope discrimination (Δ^13^C)

Carbon isotope composition was determined in milled grain samples. For each sample, 15 mg of finely milled material was weighed and analysed at the research support service of the Universidade da Coruña (Spain) using an elemental analyzer (EA1108; Carlo Erba Strumentazione, Milan, Italia) coupled to an isotope ratio mass spectrometer (Delta C; Finnigan, Mat., Bremen, Germany) operating in continuous flow mode. Values were expressed in composition units as

1 $$ \delta^{{{13}}} {\text{C }}\left( \textperthousand \right) = \left[ {\left( {{\text{R}}_{{{\text{sample}}}} /{\text{R}}_{{{\text{standard}}}} } \right) - {1}} \right] \times {1}000, $$

where the ^13^C/^12^C ratio of the sample is noted as δ^13^C and expressed in ‰, whereas R_standard_ is the molar abundance ratio of the secondary standard calibrated against the primary standard Pee Dee Belemnite (δ^13^C). The δ^13^C values were later transformed into carbon isotopic discrimination values (Δ^13^C) according to Farquhar et al.^21^ as follows:

2 $$ \Delta^{{{13}}} {\text{C }}\left( \textperthousand \right) = \left( {\delta^{{{13}}} {\text{C}}_{{{\text{air}}}} \left( \textperthousand \right) - \delta^{{{13}}} {\text{C}}_{{{\text{VPDV}}}} \left( \textperthousand \right)} \right)/\left( {{1} + \left( {\delta^{{{13}}} {\text{C}}_{{{\text{VPDV}}}} \left( \textperthousand \right)/{1}000} \right)} \right) $$

where δ^13^C_air_ (‰) is the ratio of the isotopes of ^13^C and ^12^C in the air, which varies through time, and δ^13^C_VPDV_ (‰) refers to carbon isotope discrimination of grain sample. Air δ^13^C values were obtained from Zhao et al.^19^.

#### Starch and soluble sugars concentrations

Milled grain samples were extracted by the addition of 0.5 mL of 100% ethanol then another 0.5 mL of 80% ethanol to approximately 25 mg of sample and heated in a thermomixer (70 °C, 90 min, 1100 rpm). The mixture was centrifuged (22 °C, 10 min, 14,000 rpm) and the supernatant was used for the determination of soluble sugars (glucose, fructose and sucrose). The samples were diluted with water (300 µL sample + 700 µL H_2_O Mili-Q) and measured using an ionic chromatograph (ICS-3000, Thermo Scientific Dionex, USA). Reference was made to sugar standards of known concentrations (50 mM). The pellet was used to determine the starch content. Starch was solubilized by adding KOH (0.2 N) to the pellet, and the pH was adjusted to 4.8 with acetic acid (0.1 N). The extraction was performed with the kit containing the enzyme amyloglucosidase (R-Biopharm, AG; Darmstadt, Germany) and the absorbance was measured with a spectrophotometer at 340 nm.

#### Protein content

Grain protein content (%) was determined according to Suchy et al.^22^.

#### Mineral composition

In each case, 100 mg of pulverized dry grain samples were analysed^23^. C and N concentrations (%) were determined using an elemental analyzer (EA1108; Carlo Erba Strumentazione, Milan, Italia). In addition, micro- and macro-nutrients (Cu, Zn, Fe, Mn, K, P, Mg, Ca and Na) were quantified using ICP/OES (inductively coupled plasma/optical emission spectrometry, iCAP 6500 Duo, Thermo Fisher Scientific, Waltham, USA).

### Data analyses

Given the long time-series considered, wheat samples received from herbaria were harvested from different locations and periods. Distinct genotypes were represented within each sampling year, with different number of repetitions. In view of the available data, the statistical analyses aimed to evaluate the trend of grain quality parameters over 166 years by calculating the average per year, without considering the genotype effect. To study the ‘year’ effect on the different parameters measured for global and Rothamsted grain samples, a simple analysis of variance (ANOVA) was performed by using STATGRAPHICS Centurion version 17.1.02 program (Bitstream, Cambridge, MN). For analytical variables, a multiple-range test (Fisher’s least significant differences, LSD) was applied to test for statistical differences between years. Multifactor analyses of variance and Pearson correlation analyses were performed between the different parameters and the environmental factors ([CO_2_], temperature and precipitation) with the R software (RStudio v.3.4.2, 2017; Boston-Seattle, USA). The correlations between variables were considered significant when *p* < 0.05.

## Results

### Global samples

#### Environmental conditions

Atmospheric [CO_2_] has been rising since 1850, and results in Table 2 showed that two periods can be distinguished: a first period (1850–1965), during which the average [CO_2_] slowly rose by 31 ppm in 115 years; and a second period (1965–2016) during which a swift increase of [CO_2_] of 82 ppm was recorded over 51 years. Therefore, the analysis of the effect of climate change on global wheat grain quality was based on the comparison among years, and also between these two periods (Table 3). The global mean annual temperature presented in Table 2 showed that it has increased by 1.2 °C from 1850 to 2016, with fluctuations recorded in-between.

**Table 3:** Global averages of wheat grain quality traits in [1850–1955] and [1965–2016].

Grain quality traits	[1850–1955]	[1965–2016)	*p* value
Δ^13^C (‰)	16.53 ± 0.23	16.50 ± 0.36	0.940
Starch (µmol/g DW)	2712.72 ± 117.48	2900 ± 33.11	0.344
Sucrose (µmol/g DW)	13.83 ± 2.93	15.19 ± 3.33	0.794
Glucose (µmol/g DW)	1.01 ± 0.19	1.89 ± 0.51	**0.027**
Fructose (µmol/g DW)	3.14 ± 0.59	2.92 ± 0.41	0.821
Protein content (%)	16.96 ± 1.28	13.07 ± 0.8	**0.049**
Carbon content (%)	40.81 ± 0.33	41.53 ± 0.4	0.187
C/N (%)	17.16 ± 0.56	20.6 ± 0.84	**0.039**

Mean ± standard error (SE) (n = 3–18). The calculation of *p* values is based on one-way ANOVA. Values in bold indicate statistical significance (*p* < 0.05).

#### Carbon isotope discrimination (Δ^13^C)

The results of carbon isotope discrimination calculated from 1850 to 2016 did not show a clear trend during this period (Fig. 2A). While the global carbon isotope discrimination (Δ^13^C) showed significant differences among years between 1850 and 2016 (*p* < 0.001; Fig. 2A), no significant difference was observed between 1850–1955 and 1965–2016 (16.53‰ vs. 16.50‰ respectively; Table 3). Pearson analyses did not show any significant correlation, neither between Δ^13^C and [CO_2_] nor between Δ^13^C and temperature (Table 5). Similarly, the multifactor analysis of variance (Table 6) indicated that there are no significant effects of [CO_2_] and temperature on carbon isotope discrimination, and only the interaction between these two environmental factors was statistically significant (*p* = 0.004).

#### Non-structural carbohydrates

#### Starch content

The comparison of grain starch content among years (Fig. 3A) showed significant differences between years until 1946, but since 1953, the results showed higher values compared to the previous period and stability in starch content was detected. Further, the comparison between the two periods (Table 3) showed a non-significant increase of grain starch content by 7%. Results presented in Table 5 showed non-significant negative correlations between starch content and high temperature (r = − 0.175) whereas significant positive correlation was detected with [CO_2_] (r = 0.247). The multifactor analysis of variance presented in Table 6 showed that [CO_2_] and temperature have significant effects on grain starch content, while the interaction [CO_2_] × temperature has no significant effect (*p* = 0.525).

#### Soluble sugars concentrations

Sucrose, glucose and fructose concentrations of global samples showed an increasing trend since 1975 (Fig. 4A). The comparison between the two periods (1850–1955 vs. 1965–2016; Table 3) showed non-significant differences for sucrose and fructose, whereas a significant increase was found for glucose (*p* = 0.027). Pearson analyses showed significant positive correlations between [CO_2_] and glucose and sucrose concentrations, and between temperature and glucose and fructose concentrations (Table 5). Table 5 shows that [CO_2_] had a significant effect on increasing glucose and sucrose concentrations, but no effect was recorded on fructose concentration. The interaction [CO_2_] × temperature only significantly affects sucrose concentration.

#### Protein content

Protein content has varied among years since 1850 (Fig. 5A), and the comparison between 1850–1955 and 1965–2016 revealed a significant decrease of 23% (Table 3). Significant correlations were detected between protein content and [CO_2_] and temperature (r = − 0.265 and r = 0.269, respectively; Table 5). We also detected a significant effect of [CO_2_] and temperature on protein content in the ANOVA, but no effect of the interaction effect (Table 6).

#### Mineral composition

At the global level, no significant difference was detected in C content during the period of study (Fig. 6A). However, given the decrease in protein content (that is tightly linked with the N content), the C/N ratio showed a significant increase of 20% between 1850–1955 and 1965–2016 (Fig. 7A, Table 3). A significant positive correlation was found between [CO_2_] and C/N ratio, as well as a significant effect of [CO_2_] on this ratio in the ANOVA (Tables 5, 6). On the contrary, a significant negative correlation was found between the temperature and the C/N ratio (and a significant effect of the temperature in the ANOVA; Tables 5, 6).

The global analyses of macro/micro-elements showed that their concentrations tend to decrease as ambient [CO_2_] increased (Fig. 8A). In fact, negative correlations were detected between all macro/micro-elements and [CO_2_], and these correlations were significant (and the effect of [CO_2_] significant in the ANOVA) for K, Mg, Zn, Fe and Mn (Table 5). The magnitude of the reduction differed between minerals, and the most notable reductions were observed for Mn, Fe, Zn and Mg (Fig. 8A; Table 6). Temperature has also significantly affected Mg, Fe and Mn concentrations, but no interaction with [CO_2_] was detected (except for Cu; Table 6).

### Broadbalk Wheat experiment (Rothamsted, UK)

#### Environmental conditions

Since the beginning of the Broadbalk wheat experiment in Rothamsted, the [CO_2_] concentration increased by 45 ppm from 1850 to 1975 (i.e., in 125 years), and by as much as 47 ppm from 1975 to 2016 (i.e., in 41 years; Table 2). Annual temperatures were recorded from 1878 to 2016. Available data showed that, while mean temperature during 1878 was of 9.3 °C, during, 2016, this value reached 10.3 °C. Precipitation data recorded between 1878 and 2016 in Rothamsted did not show a clear pattern over time and rather revealed random fluctuations among years (Table 2).

#### Grain yield and thousand kernel weight (TKW)

**Figure 1:**
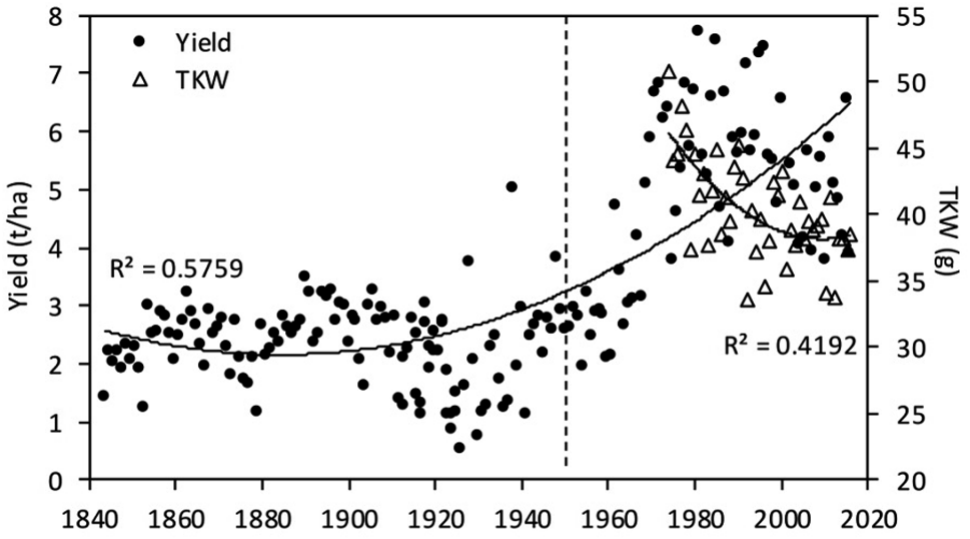
Trends in wheat yield and thousand kernel weight (TKW) of Broadbalk experiment from 1850 to 2016. Data are means. The dashed line represents the introduction of dwarf cultivars in 1968.

The results provided by the Broadbalk experiment about grain yield and thousand kernel weight (TKW) are presented in Fig. 1. Grain yield was more or less constant from 1850 until 1960, but has been subsequently increasing. Correlation analyses revealed significant positive correlations between grain yield and precipitation, [CO_2_] and temperature (Tables 4, 5). Highly significant effects of [CO_2_] and temperature on wheat yield were also observed (Table 6). Further, available data showed that TKW has been decreasing since 1974 (Fig. 1). Pearson analysis revealed non-significant correlation between TKW and grain yield (Table 4), and the multifactor ANOVA showed highly significant effects of [CO_2_] and temperature on TKW (Table 6).

**Table 4:** Pearson correlation analyses (r) between environmental parameter (precipitation), and grain yield and quality traits of the Rothamsted Broadbalk experiment.

Parameters	Yield	Starch	Sucrose	Glucose	Fructose	Protein
Precipitation	0.662***	− 0.198	0.207	0.126	− 0.310	0.004
TKW	0.193	–	–	–	–	–

A statistically significant effect is indicated with *** for *p* < 0.001. ‘–’ indicates unavailable coefficient of correlation (r).

**Table 5:** Pearson correlation analyses (r) between grain yield and quality traits, and environmental factors ([CO_2_] and temperature).

Grain quality traits	Global		Rothamsted	
	[CO_2_]	Temperature	[CO_2_]	Temperature
Yield	–	–	0.631**	0.688***
Δ^13^C	0.048	0.003	− 0.688***	0.143
Starch	0.247*	− 0.175	0.458*	0.435*
Glucose	0.379**	0.238*	0.591**	0.755***
Sucrose	0.676***	− 0.03	0.209	− 0.424
Fructose	0.131	0.257*	− 0.224	0.293
Protein content	− 0.265**	0.269**	− 0.771***	− 0.474*
C/N ratio	0.229**	− 0.396***	0.691***	0.614**
C	− 0.120	0.107	0.482*	0.315
K	− 0.452***	− 0.151	0.486*	0.425*
P	− 0.124	0.036	− 0.860***	− 0.514*
Ca	− 0.037	0.202*	− 0.391	− 0.626**
Mg	− 0.560***	0.169	− 0.852***	− 0.698***
Cu	− 0.165	− 0.116	− 0.437*	− 0.370
Na	0.054	0.080	− 0.324	− 0.557**
Zn	− 0.285*	0.097	− 0.717***	− 0.346
Fe	− 0.188*	0.212*	− 0.314	− 0.300
Mn	− 0.348***	0.364***	− 0.942***	− 0.444*

Statistically significant effects are indicated with *** for *p* < 0.001, ** for *p* < 0.01 and * for *p* < 0.05. ‘–’ indicates unavailable coefficient of correlation (r).

**Table 6:** Multifactor analysis of variance (ANOVA).

Traits	Global			Rothamsted		
	[CO_2_]	Temperature	[CO_2_] × temperature	[CO_2_]	Temperature	[CO_2_] × temperature
	*p* value			*p* value		
Yield	**–**	**–**	–	**< 0.001**	**0.003**	**0.015**
TKW	**–**	**–**	–	**< 0.001**	**< 0.001**	0.164
Δ^13^C	0.612	0.906	**0.004**	**< 0.001**	**< 0.001**	0.837
Starch	**0.009**	**0.008**	0.525	**0.026**	0.247	0.700
Glucose	**0.001**	0.079	0.249	**< 0.001**	**< 0.001**	**< 0.001**
Sucrose	**< 0.001**	0.215	**0.002**	0.147	< 0.001	0.806
Fructose	0.273	**0.023**	0.962	0.233	**0.002**	0.434
Protein content	**< 0.001**	**< 0.001**	0.901	**< 0.001**	0.737	**0.015**
C/N ratio	**0.001**	**< 0.001**	0.064	**< 0.001**	0.060	0.794
C	0.137	0.093	0.158	**0.022**	0.717	0.852
K	**< 0.001**	0.446	**0.001**	**0.016**	0.296	0.318
P	0.174	0.488	0.248	**< 0.001**	0.538	0.700
Ca	0.682	0.018	0.552	**0.035**	**0.010**	0.857
Mg	**< 0.001**	**< 0.001**	0.264	**< 0.001**	**0.004**	0.151
Cu	0.065	0.345	**0.027**	**0.038**	0.416	0.849
Na	0.559	0.444	0.755	0.084	**0.019**	0.218
Zn	**0.013**	0.498	0.528	**< 0.001**	0.809	0.845
Fe	**0.035**	**0.047**	0.273	0.141	0.448	0.340
Mn	**< 0.001**	**< 0.001**	0.586	**< 0.001**	0.298	**0.002**

The calculation of *p* values in based on multifactor analysis of variance ANOVA. Values in bold indicate significance (*p* < 0.05). ‘–’ indicates unavailable *p* value.

#### Carbon isotope discrimination (Δ^13^C)

**Figure 2:**
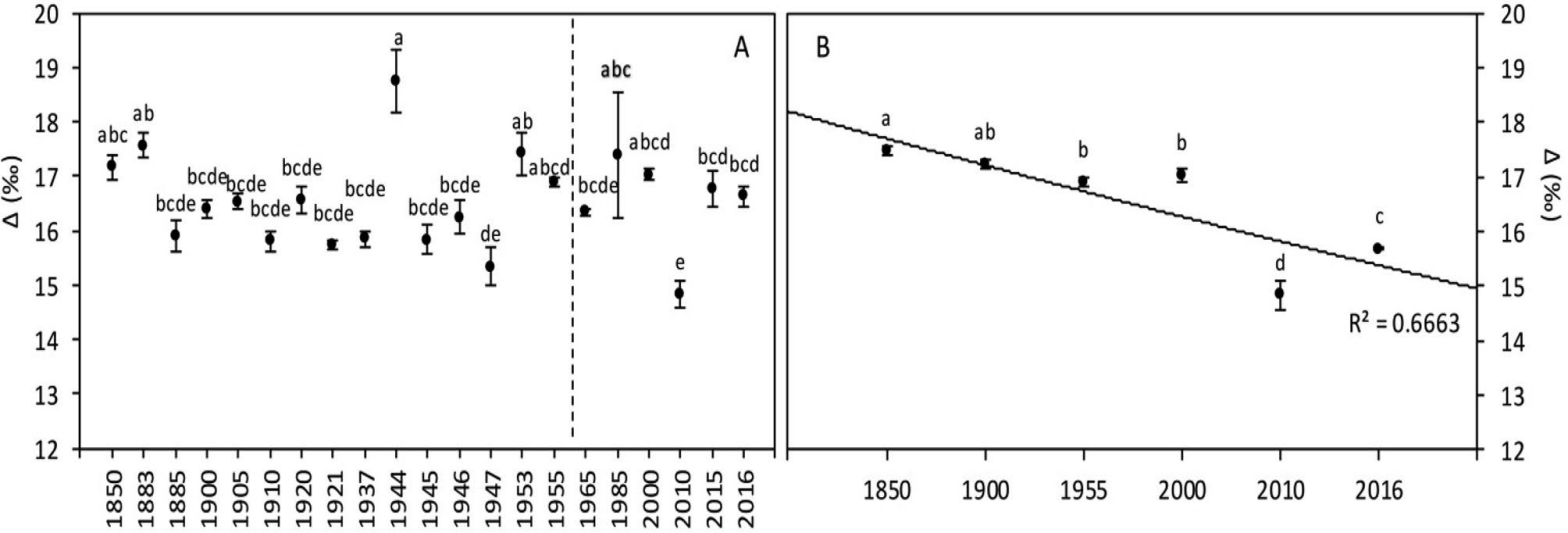
Wheat grain carbon isotope discrimination (Δ^13^C) of global (**A**) and Broadbalk experiment (**B**) samples. The dashed line corresponds to the separation between the [1850–1955] and [1965–2016] periods, based on the increase of [CO_2_]. Data are means ± standard errors (n = 3–14). The same letters indicate no statistically significant differences among years (Fisher’s LSD, *p* ≥ 0.05).

We observed a clear decrease of Δ^13^C during the last decades (Fig. 2B). The [CO_2_] concentration was highly significantly and negatively correlated with Δ^13^C (Table 5), and had a significant effect on this variable (Table 6). The temperature has also significantly affected Δ^13^C, but there was no significant effect of the [CO_2_] × temperature interaction.

#### Non-structural carbohydrates

#### Starch content

**Figure 3:**
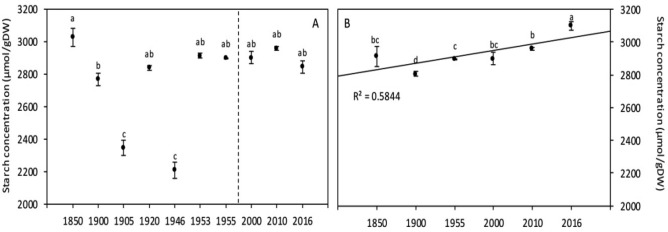
Wheat grain starch content of global (**A**) and Broadbalk experiment (**B**) samples. The dashed line corresponds to the separation between the [1850–1955] and [1965–2016] periods, based on the increase of [CO_2_]. Data are means ± standard errors (n = 3–14). The same letters indicate no statistically significant differences among years (Fisher’s LSD, *p* ≥ 0.05).

Grain starch content varied significantly among years since 1850 and reached the highest value in 2016 (Fig. 3B). It should also be noted that at Rothamsted, the increasing temperature might have contribute to favor starch accumulation (*p* = 0.034) by 43% (Table 5), but there was no correlation between precipitation and starch content (Table 4). Results presented in Table 4 showed insignificant negative correlations between starch content and precipitations (r = − 0.198) whereas significant correlations have been recorded with [CO_2_] and temperature (Table 5). According to the results presented in Table 6, only raising [CO_2_] has a significant effect on grain starch content since 1850 to 2016.

#### Soluble sugars concentrations

**Figure 4:**
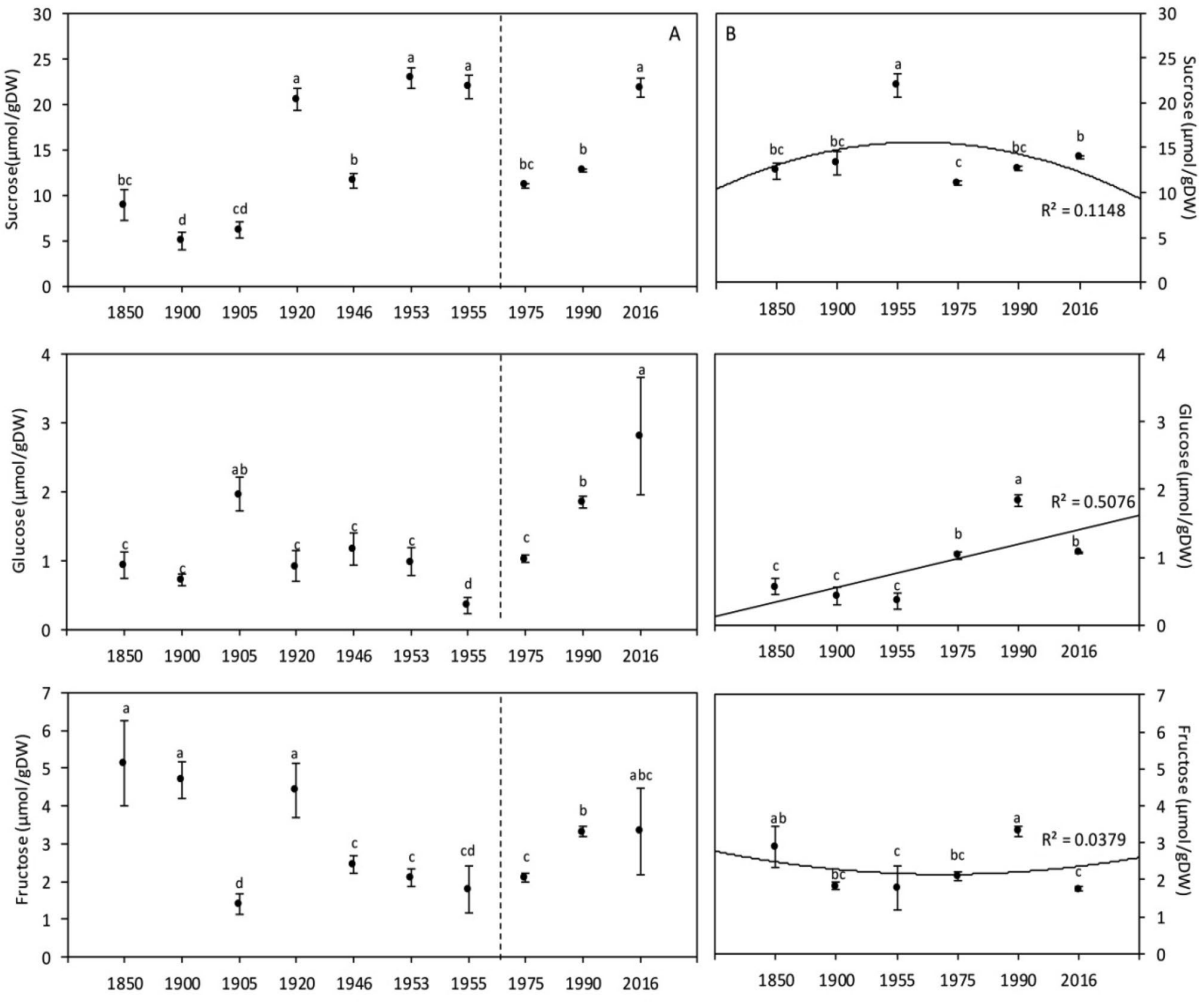
Wheat grain soluble sugar (sucrose, glucose and fructose) concentration of global (**A**,**C**,**F**) and Broadbalk experiment (**B**,**D**,**G**) samples. The dashed line corresponds to the separation between the [1850–1955] and [1975–2016] periods, based on the increase of [CO_2_]. Data are means ± standard errors (n = 3–14). The same letters indicate no statistically significant differences among years (Fisher’s LSD, *p* ≥ 0.05).

The concentrations of sucrose, glucose and fructose in wheat grains showed significant variations among years, as presented in Fig. 4B. Pearson analyses showed non-significant correlations between these concentrations and precipitation (Table 4). [CO_2_] and temperature were significantly related to glucose concentration, as shown by both the positive correlations (r = 0.591 and r = 0.755, respectively; Table 5) and the ANOVA (Table 6).

#### Protein content

**Figure 5:**
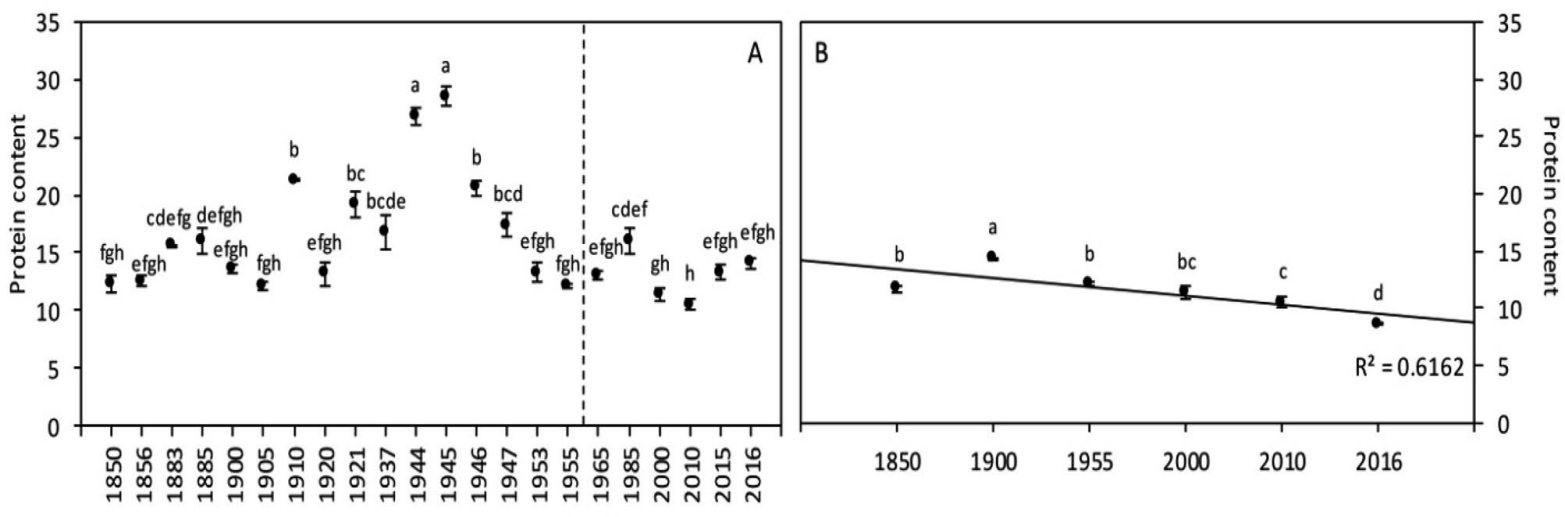
Wheat grain protein content of global (**A**) and Broadbalk experiment (**B**) samples. The dashed line corresponds to the separation between the [1850–1955] and [1965–2016] periods, based on the increase of [CO_2_]. Data are mean ± standard errors (n = 3–14). The same letters indicate no statistically significant differences among years (Fisher’s LSD, *p* ≥ 0.05).

Climate change has negatively impacted total protein content: A 26% reduction in protein content was recorded between 1850 and 2016 (Fig. 5B). Pearson analyses revealed that water availability was not correlated with protein content (Table 4), whereas temperature and (more significantly) [CO_2_] were both negatively correlated with such content (Table 5). Based on the ANOVA, only [CO_2_] but not temperature had a significant effect on protein content (Table 6).

#### Mineral composition

**Figure 6:**
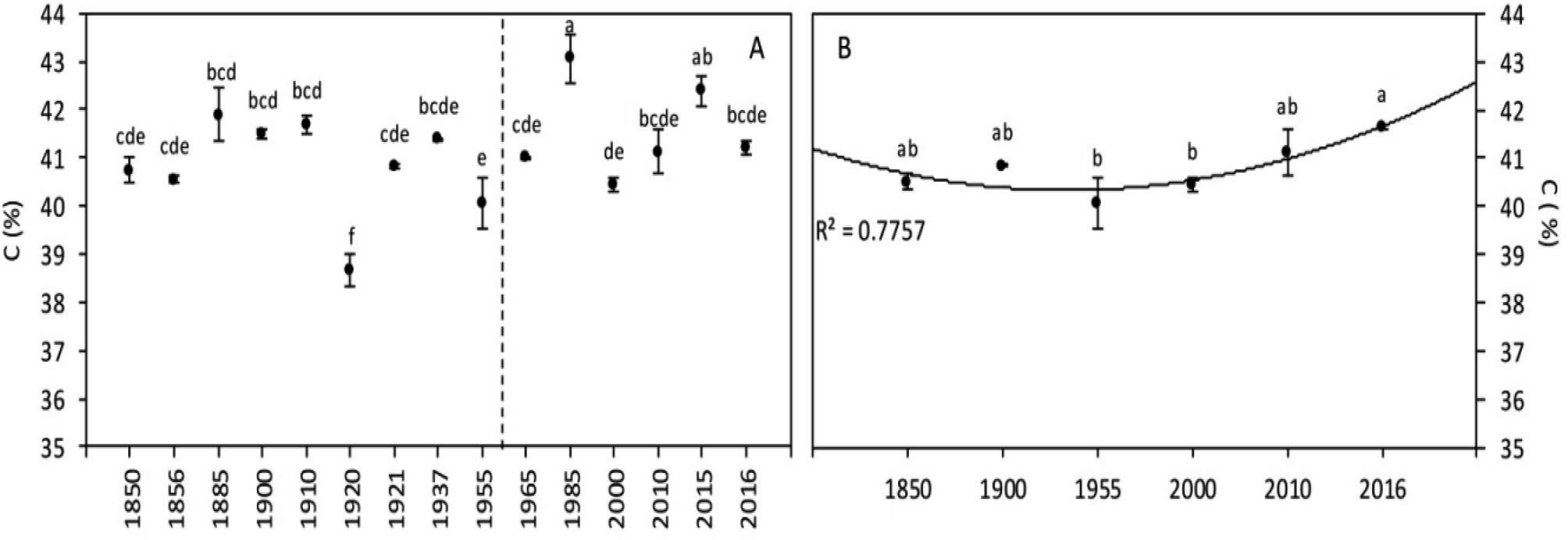
Wheat grain carbon content of global (**A**) and Broadbalk experiment (**B**) samples. The dashed line corresponds to the separation between the [1850–1955] and [1965–2016] periods, based on the increase of [CO_2_]. Data are means ± standard errors (n = 3–14). The same letters indicate no statistically significant differences among years (Fisher’s LSD, *p* ≥ 0.05).

**Figure 7:**
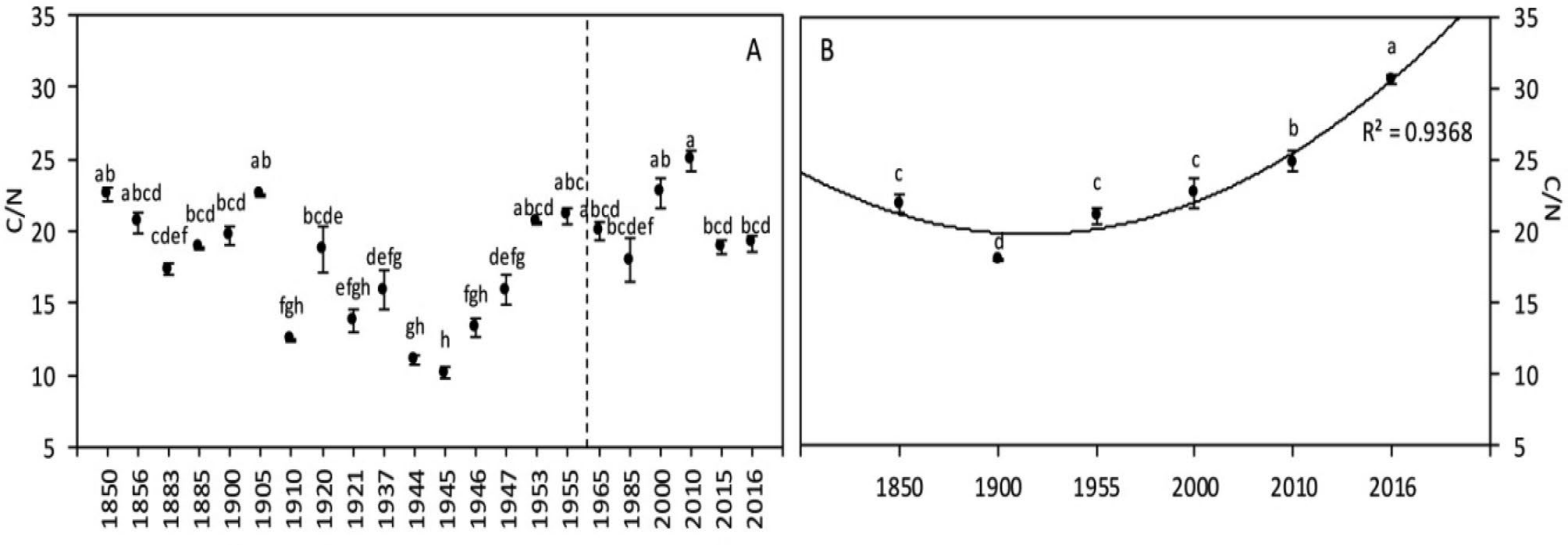
Wheat grain C/N ratio of global (**A**) and Broadbalk experiment (**B**) samples. The dashed line corresponds to the separation between the [1850–1955] and [1965–2016] periods, based on the increase of [CO_2_]. Data are means ± standard errors (n = 3–14). The same letters indicate no statistically significant differences among years (Fisher’s LSD, *p* ≥ 0.05).

Carbon content (Fig. 6B) showed a significant 3% increase since 1850. Similarly, C/N ratio (Fig. 7B) showed a highly significant 40% increase, mainly caused by the significant decrease of protein content (and therefore N content) reported above. Only [CO_2_], but not the temperature, was significantly correlated with and had a significant effect on C content (and consequently on C/N ratio; Tables 5, 6).

**Figure 8:**
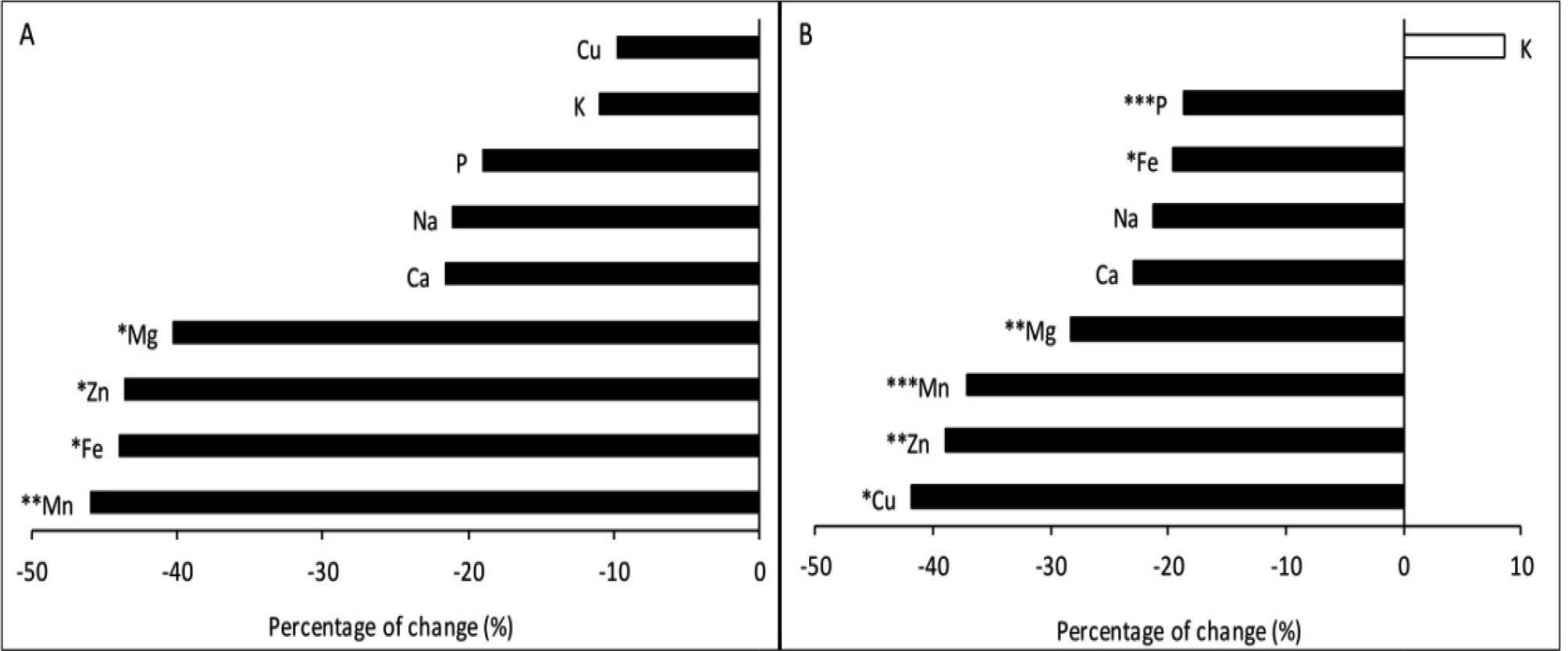
Change in grain minerals concentration relative to [1850–1955] period of global (**A**) and Broadbalk experiment (**B**) samples. Data are means ± standard errors (n = 3–14). Statistically significant effects are indicated with *** for *p* < 0.001, ** for *p* < 0.01 and * for *p* < 0.05 (Fisher’s LSD).

Macro/micro-elements concentrations have also been modified since 1850 (Fig. 8B). Statistical analyses showed that both [CO_2_] and temperature (but most importantly [CO_2_]) had significant negative effects on mineral compositions (except for the K concentration that increased; Tables 5, 6).

## Discussion

The current study, based on historical wheat samples collected over a 166-year period, has confirmed that grain quality and yield have been affected by raising atmospheric [CO_2_] and temperature. The increase of [CO_2_] and temperature, together with the introduction of dwarfing genotypes (see below), have increased harvest index, leading to rising wheat yield. Furthermore, carbon isotope discrimination has decreased over 166 years in the Broadbalk experiment (but not on worldwide samples collected across the same time period), which indicates that plants have been subjected to stressful conditions causing stomatal closure. With respect to grain quality parameters, our findings showed that non-structural carbohydrate concentrations have increased, while total protein content and mineral compositions have decreased.

The decreasing trend in TKW indicated that the increase in grain yield was not linked to heavier grains, but to a larger number of grains. While the effect of introducing semi-dwarf varieties should also be considered, our study showed that increasing [CO_2_] favored crop production. Previous experiments, carried out with wheat plants grown in environments where atmospheric [CO_2_] was increased by 150–300 ppm, showed similar increases in grain yield values^5,24^. As has been widely described in these previous experiments, the higher yield records would be associated with the stimulation of photosynthetic rates. According to this study, alongside increasing radiation levels, the temperature rise was also an important factor explaining yield increases. More specifically, the enhancement of ambient temperatures (below heat stress values) might have contributed to extend the grain filling period, which subsequently resulted in a higher biomass production and a higher yield. Finally, when analyzing changes in crop yield during the last decades, together with increasing [CO_2_] and temperature effects, changes in crop varieties could have an important effect. After 1968, high-yielding semi-dwarf cultivars (with increasing harvest index) were cultivated in the Rothamsted field trials and these cultivars have been reportedto distribute a greater proportion of photosynthates to the grains than other cultivars^25^.

The use of stable isotope variation has grown steadily in plant research during the past two decades. Stable isotopes are time-integrated indicators of how plants interact and respond to their abiotic and biotic environments^26^. Consequently, alterations in Δ^13^C have been used as a selection indicator of stomatal opening, water transpiration and water use efficiency (WUE) under different water availability and [CO_2_]^5,26^. In the case of Rothamsted, the negative correlation between [CO_2_] and Δ^13^C detected after the 1960’s revealed that following the increase in [CO_2_] and temperature, and the decrease of precipitation, plants tended to decrease stomatal opening and reduce water loss via canopy transpiration^19^. As described by Aranjuelo et al.^27^, exposure to elevated [CO_2_] might increase WUE by decreasing water consumption (due to a decline in stomatal opening and transpiration), by enhancing CO_2_ fixation, or by the interaction of both mechanisms. However, large genetic variation in carbon isotope discrimination exists among and between species. This variation could be widely explained by genotypic differences in stomatal conductance and photosynthetic capacity^28^. Hence, the absence of a temporal trend at the global (worldwide) level, between 1850 and 2016, is probably mainly due to genotypic variability of cultivars grown at different locations.

About the carbohydrate content, studies conducted in controlled and FACE facilities showed that rising [CO_2_] contributes to increases in starch and soluble sugar contents in wheat grains^29–31^. The larger photosynthetic rates of C_3_ plants such as wheat under increasing [CO_2_] may contribute to increased carbohydrate translocation from the source (leaves and stems) to the sink (grains), where the C is mainly stored in the form of starch. Furthermore, the positive correlation between yield and temperature at Rothamsted contributed to increased starch content. Considering that photosynthesis increases as leaf temperature rises (peaking at an optimum temperature and then declining), and that average temperature values in this area are below the optimum (15–25 °C), such an increase in ambient temperature might have contributed to increase photosynthesis and grain development^32,33^. Increases of ambient temperatures has been previously described and often results in an environmental temperature closer to the optimal, which results in increased photosynthesis^34,35^.

Alongside the increases in grain carbohydrates mentioned above, decreases in total protein and nutrient concentrations have been extensively described in plants exposed to elevated [CO_2_]^13,36^. Different explanations have been proposed. According to some studies^16,37^, decreased protein and mineral concentrations could be a consequence of the larger carbohydrate content in grains. Our study showed a clear correlation between [CO_2_] and C/N ratio in all cases. Furthermore, we found that the starch concentration globally increased by 7% while grain protein content decreased by 23% between 1850–1955 and 1965–2016. This may support the fact that grain protein decrease should also be associated with other mechanisms such as decreased transpiration-driven mass flow of nitrogen^38,39^. Furthermore, other factors such as the crop breeding approaches aimed at increasing crop yields (and declining response to N fertilizer) and limitations to N assimilation^40^ may have been involved. The positive correlation found between temperature and starch content in the Broadbalk experiment, as well as changes to the C/N ratio, would also highlight how the temperature-associated increases in grain C content are involved in the lower grain protein contents observed.

The current study showed an overall decrease in all micro- and macronutrient concentrations in wheat grain over 166 years. Similarly, other historical studies have shown that mineral composition of the dry matter of wheat grains, vegetables and some fruits have decreased over time^6,41–43^. Such impoverishment may in part be associated with changes in atmospheric [CO_2_] and temperature. The fact that the decreases were more evident in the Broadbalk experiment could be associated with different factors such as the strong increases in crop yield and lower ^13^C discrimination (Δ) values detected in those plants during the recent decades. The potential impact of other factors such as alterations on nutrient uptake, and remobilization from leaves to grain and a greater transport of carbohydrates to grain (dilution) should also be considered. In addition, lower stomatal opening and a decrease of crop transpiration may have altered the mass flow of minerals from the soil to aboveground plant parts^44,45^. As observed by Fan et al.^6^, this explanation is especially likely since the concentrations of soil nutrients have not decreased at Rothamsted since the Green Revolution.

## Conclusions and perspectives

Overall, this study highlighted that there has been a global trend of altered wheat grain quality characterized by an increase in non-structural carbohydrates and an impoverishment in total protein and mineral nutrients concentrations during the last 166 years. This trend has been especially prominent since the 1960s and linked to the introduction of higher yielding short-strawed varieties, together with an increase in air [CO_2_] and temperature. It seems likely that during this period enhanced photosynthetic rates linked to the increase in [CO_2_] may have favored carbohydrate synthesis and carbon accumulation in grains and that this has negatively affected mineral composition. Along with a potential C-derived dilution effect, the current paper also implicates other factors such as depleted transpiration (affecting mineral transport) and the lower responsiveness of modern cultivars to current fertilization strategies. In view of the findings stated above, breeding strategies should develop new genotypes better adapted to changing environmental conditions with greater resource use efficiency and combine high grain nutritional values with high-yielding traits by exploring genetic variation in proteins and nutrients concentrations in wheat germplasm, since these traits are not only affected by environmental factors, but also are controlled genetically.

## Supplementary information

Supplementary Table.

## Data Availability

The datasets analyzed during the current study are available from the corresponding author on reasonable request.

## References

[1] Scheelbeek PFD (2018). Effect of environmental changes on vegetable and legume yields and nutritional quality. PNAS.

[2] Intergovernmental Panel on Climate Change (IPCC). Climate change 2014: Synthesis report. In *Contribution of Working Groups I, II and III to the Fifth Assessment Report of the Intergovernmental Panel on Climate Change* (eds. Core Writing Team, Pachauri, R.K. & Meyer, L.A.) (IPCC, Geneva, 2014). https://www.ipcc.ch/report/ar5/syr/.

[3] European Environment Agency (EEA). (Accessed January 10, 2019) https://www.eea.europa.eu/.

[4] Oury FX (2012). A study of genetic progress due to selection reveals a negative effect of climate change on bread wheat yield in France. Eur. J. Agron..

[5] Erice G (2019). Impact of elevated CO_2_ on yield and quality traits of a historical (Blanqueta) and a modern (Sula) durum wheat. J. Cereal Sci..

[6] Fan MS (2008). Evidence of decreasing mineral density in wheat grain over the last 160 years. J. Trace Elem. Med. Biol..

[7] Nirgude RR, Sonawane KG (2017). An estimation of impact of wheat production technology. Trends Biosci..

[8] Degener JF (2015). Atmospheric CO_2_ fertilization effects on biomass yields of 10 crops in northern Germany. Front. Environ. Sci..

[9] Kant S (2012). Improving yield potential in crops under elevated CO_2_: Integration the photosynthetic and nitrogen utilization efficiencies. Front. Plant Sci..

[10] DaMatta FM, Grandis A, Arenque BC, Buckeridge MS (2010). Impacts of climate changes on crop physiology and food quality. Food Res. Int..

[11] Taub DR, Miller B, Allen H (2008). Effects of elevated CO_2_ on the protein concentration of food crops: A meta-analysis. Glob. Change Biol..

[12] Högy P, Fangmeier A (2008). Effects of elevated atmospheric CO_2_ on grain quality of wheat. J. Cereal Sci..

[13] Loladze I (2014). Hidden shift of the ionome of plants exposed to elevated CO_2_ depletes minerals at the base of human nutrition. eLife.

[14] Körner C (2006). Plant CO_2_ responses: An issue of definition, time and resource supply. New Phytol..

[15] Morales F (2014). Methodological advances: Using greenhouses to stimulate climate change scenarios. Plant Sci..

[16] Högy P (2009). Effects of elevated CO_2_ on grain yield and quality of wheat: Results from a 3-year free-air CO_2_ enrichment experiment. Plant Biol..

[17] Pinkard EA, Beadle CL, Mendham DS, Carter J, Glen M (2010). Determining photosynthetic responses of forest species to elevated [CO_2_]: Alternatives to FACE. For. Ecol. Manag..

[18] Pinter PJ (2000). Free air CO_2_ enrichment (FACE): Blower effects on wheat canopy microclimate and plant development. Agric. For. Meteorol..

[19] Zhao FJ, Spiro B, McGrath SP (2001). Trends in C_13_/C_12_ ratios and C isotope discrimination of wheat since 1875. Oecologia.

[20] Intergovernmental Panel on Climate Change (IPCC). Climate change 2007: Synthesis report. In *Contribution of Working Groups I, II and III to the Fourth Assessment Report of the Intergovernmental Panel on Climate Change* (eds. Core Writing Team, Pachauri, R.K. & Reisinger, A.) (IPCC, Geneva, 2007). https://www.ipcc.ch/report/ar4/syr/.

[21] Farquhar GD, Ehleringer JR, Hubick KT (1989). Carbon isotope discrimination and photosynthesis. Annu. Rev. Plant Physiol. Plant Mol. Biol..

[22] Suchy J (2007). Rapid assessment of glutenin and gliadin in wheat by UV spectrophotometer. Crop Sci..

[23] Gámez AL, Soba D, Zamarreño AM, García-Mina JM, Aranjuelo I, Morales F (2019). Effect of water stress during grain filling on yield, quality and physiological traits of Illpa and Rainbow Quinoa (*Chenopodium quinoa* Willd.) cultivars. Plants.

[24] Högy P, Zörb C, Langenkämper G, Betsche T, Fangmeier A (2009). Atmospheric CO_2_ enrichment changes the wheat grain proteome. J. Cereal Sci..

[25] Flintham JE, Borner A, Worland AJ, Gale MD (1997). Optimizing wheat grain yield: Effects of Rht (gibberellin-insensitive) dwarfing genes. J. Agric. Sci..

[26] Yousfi S, Serret MD, Araus JL (2013). Comparative response of δ^13^C, δ^18^O and δ^15^N in durum wheat exposed to salinity at the vegetative and reproductive stages. Plant Cell Environ..

[27] Aranjuelo I, Irigoyen JJ, Perez P, Martinez-Carrasco R, Sanchez-Díaz M (2005). The use of temperature gradient tunnels for studying the combined effect of CO_2_, temperature and water availability in N_2_ fixing alfalfa plants. Ann. Appl. Biol..

[28] Dixon LS, Godoy JV, Carter AH (2019). Evaluating the utility of carbon isotope discrimination for wheat breeding in the pacific northwest. Plant Phenomics.

[29] Sinha PG, Saradhi PP, Uprety DC, Bhatnagar AK (2011). Effect of elevated CO_2_ concentration on photosynthesis and flowering in three wheat species belonging to different ploidies. Agric. Ecosyst. Environ..

[30] Pandey V (2017). Impact of elevated CO_2_ on wheat growth and yield under free air CO_2_ enrichment. J. Sci. Res..

[31] Yadav A, Bhatia A, Yadav S, Kumar V, Singh B (2019). The effects of elevated CO_2_ and elevated O_3_ exposure on plant growth, yield and quality of grains of two wheat cultivars grown in north India. Heliyon.

[32] Nuttall JG (2017). Models of grain quality in wheat—A review. Field Crop Res..

[33] Posch BC (2019). Exploring high temperature responses of photosynthesis and respiration to improve heat tolerance in wheat. J. Exp. Bot..

[34] Albert KR, Mikkelsen TN, Michelsen A, Ro-Poulsen H, Van der Linden L (2011). Interactive effects of drought, elevated CO_2_ and warming on photosynthetic capacity and photosystem performance in temperate heath plant. J. Plant Physiol..

[35] Sage RF, Kubien DS (2007). The temperature response of C_3_ and C_4_ photosynthesis. Plant Cell Environ..

[36] Zhu C (2018). Carbon dioxide (CO_2_) levels this century will alter the protein, micronutrients, and vitamin content of rice grains with potential health consequences for the poorest rice-dependent countries. Sci. Adv..

[37] Kimball BA (2001). Elevated CO_2_, drought and soil nitrogen effects on wheat grain quality. New Phytol..

[38] Myers SS (2014). Increasing CO_2_ threatens human nutrition. Nature.

[39] Uddling J, Broberg MC, Feng Z, Pleijel H (2018). Crop quality under rising atmospheric CO_2_. Plant Biol..

[40] Vicente R (2015). Quantitative RT–PCR platform to measure transcript levels of C and N metabolism-related genes in durum wheat: Transcript profiles in elevated [CO_2_] and high temperature at different levels of N supply. Plant Cell Physiol..

[41] Garvin DF, Welch RM, Finley JW (2006). Historical shifts in the seed mineral micronutrient concentration of US hard red winter wheat germplasm. J. Sci. Food Agric..

[42] Morgounov AI (2013). Historical changes in grain yield and quality of spring wheat varieties cultivated in Siberia from 1900 to 2010. Can. J. Plant sci..

[43] White PJ, Broadley MR (2005). Biofortifying crops with essential mineral elements. Trend Plant Sci..

[44] Pilbeam DJ (2015). Breeding crops for improved mineral nutrition under climate change conditions. J. Exp. Bot..

[45] Wang Y, Frei M (2011). Stressed food—The impact of abiotic environmental stresses on crop quality. Agric. Ecosyst. Environ..

