# Supplementary material for: Assessing the evolution of wheat grain traits during the last 166 years using archived samples
Source: Sci Rep. 2020 Dec 11;10:21828. doi: 10.1038/s41598-020-78504-x (PMC7733497; doi:10.1038/s41598-020-78504-x)
Supplement: Supplementary file 1 — Supplementary Table. [file 41598_2020_78504_MOESM1_ESM.docx]

**Assessing the evolution of wheat grain traits during the last 166 years using archived samples**

Sinda Ben Mariem^1^, Angie L. Gámez^1^, Luis Larraya^2^, Teresa Fuertes-Mendaizabal^3^, Nuria Cañameras^4^, José L. Araus^5^, Steve P. McGrath^6^, Malcolm J. Hawkesford^7^, Carmen Gonzalez Murua^3^, Myriam Gaudeul^8^, Leopoldo Medina^9^, Alan Paton^10^, Luigi Cattiveli^11^, Andreas Fangmeier^12^, James Bunce^13^, Sabine Tausz-Posch^14^,Andy J. Macdonald^6^ and Iker Aranjuelo^1*^

^1^Spanish National Research Council (CSIC)-Government of Navarre, AgroBiotechnology Institute (IdAB), 31006, Av. Pamplona 123, Mutilva, Spain

^2^Institute for Multidisciplinary Applied Biology, Dpto. Agronomía, Biotecnología y Alimentación, Universidad Pública de Navarra, Campus Arrosadia, 31006 Pamplona, Spain

^3^Department of Plant Biology and Ecology, University of the Basque Country (UPV/EHU), Bilbao, Spain

^4^Universitat Politècnica de Catalunya, Esteve Terrades 8, Building 4, Castelldefels, Spain.

^5^Integrative Crop Ecophysiology Group, Plant Physiology Section, Faculty of Biology, University of Barcelona, Barcelona, and AGROTECNIO Center, Lleida, Spain.

^6^Sustainable Agriculture Sciences, Rothamsted Research, Harpenden, Hertfordshire AL5 2JQ, United Kingdom

^7^Plant Sciences, Rothamsted Research, Harpenden, Hertfordshire AL5 2JQ, United Kingdom

^8^L’Institut de Systématique, Évolution, Biodiversité, 12-16 rue Buffon, CP39, 75005 Paris

^9^Spanish National Research Council (CSIC), Real Jardín Botánico, C/ Claudio Moyano 1, Spain

^10^Royal Botanic Gardens, Kew Richmond, TW9 3AB, United Kingdom

^11^Agricultural Research Council (CREA), Fiorenzuolad’Arda, Italy

^12^Institute of Landscape and Plant Ecology, University of Hohenheim, August-von-Hartmann-Str. 3, 70599 Stuttgart, Germany

^13^Adaptive Cropping Systems Lab (retired), Beltsville Agricultural Research Center, Agricultural Research Service, US Department of Agriculture, Beltsville, MD 20705, USA

^14^Department of Agriculture, Science and the Environment, School of Health, Medical and Applied Sciences, CQ University Australia Rockhampton, QLD, Australia

Table S1. Winter wheat varieties grown on Broadbalk during 1849-2018.
